# Dietary treatment of congenital chylothorax with skimmed breast milk

**DOI:** 10.1186/s13052-021-01125-1

**Published:** 2021-08-26

**Authors:** Michaela Höck, Alexander Höller, Marlene Hammerl, Karina Wechselberger, Jakob Krösslhuber, Ursula Kiechl-Kohlendorfer, Sabine Scholl-Bürgi, Daniela Karall

**Affiliations:** 1 Department of Paediatrics II, Neonatology, Medical University of Innsbruck, Innsbruck, Austria; 2 Service for Nutrition and Dietetics, Medical University of Innsbruck, Innsbruck, Austria; 3 Department of Paediatrics I, Intensive Care Unit, Medical University of Innsbruck, Innsbruck, Austria; 4 Department of Paediatrics I, Inherited Metabolic Disorders, Medical University of Innsbruck, Anichstrasse 35, 6020 Innsbruck, Austria

**Keywords:** Congenital chylothorax, Skimmed breast milk

## Abstract

**Background:**

Congenital chylothorax (CC) is a rare but potentially life-threatening condition in newborns. It is defined as an accumulation of chyle in the pleural cavity. The few publications regarding medical management and therapeutic dietary intervention motivated us to share our experience.

**Methods:**

Neonates diagnosed with congenital chylothorax and treated at Innsbruck Medical University Hospital between 2013 and 2020 (*n* = 6, gestational age: 36 3/7, 32 5/7, 36 4/7, 35 0/7, 35 4/7, 37 3/7 weeks) were eligible for this report.

The cornerstones of treatment for chylothorax conventionally consist of chest tube drainage (CTD), respiratory support, dietary restriction of long-chain triglycerides (LCT) or total parenteral nutrition (TPN). In further course the introduction of a medium-chain triglyceride (MCT)-based formula followed by an overlapping switch to a formula with low LCT and high MCT, containing the essential long-chain fatty acids (LCFA), is attempted. In three patients we used fat-modified (skimmed) breast milk to provide a high protein and low fat diet and to avoid the discontinuation of breast milk.

**Results:**

The outcome of an early introduction of LCFA in the form of skimmed breast milk after resolution of chylothorax diverse. One patient had a favourable outcome, meaning no recurrence of pleural effusion, adequate weight gain and a content mother, while another patient had a relapse of pleural effusion after the administration of skimmed milk and was therefore transitioned back to Basic F® .

The CC of patient 5 was difficult due to Noonan syndrome. Two weeks after the introduction of skimmed breast milk the mother wanted to stop to express breast milk, so nutrition was changed to Basic F®.

**Conclusion:**

The first-line therapy of chylothorax is a combination of respiratory stabilization and dietary modification. The use of skimmed breast milk is advisable in CC and feasible by means of a simple milk defatting procedure. It offers benefits to mothers who wish to resume breast feeding after resolution of chylothorax and has proven positive effects, above all in preterm infants as optimal nutrition with protective components superior to formula feeding. However, the nutritional analysis of the skimmed milk and the correlation to a re-accumulation of pleural fluid remains a question to be answered.

## Background

Congenital chylothorax, the accumulation of chyle (lymphatic fluid of intestinal origin) in the pleural space, is a rare – 1:10.000 live births – a potentially life-threatening condition which  requires multimodal management strategies [1].

Treatment is typically stepwise, starting with stabilization of the respiratory situation via chest tube drainage (CTD) and respiratory support. Nutrition management for chylothorax includes adhering to a regime where the fat source is primarily medium-chain triglyceride (MCT). As breast milk (BM) contains high concentrations of long-chain triglycerides (LCT), patients are usually transitioned to an MCT-containing formula like Monogen®, which is a milk protein-based powdered formula with low LCT (16%) and high MCT (84%) containing the essential fatty acids docosahexaenoic acid (DHA) and arachidonic acid (AA) [2]. Alternatively, Basic F®, an almost fat-free (< 0.07 g/100 ml) cow’s milk substitute would be possible. MCTs are transported directly into the portal circulation, contributing little to chylomicron formation and minimizing the volume of lymph flowing along the thoracic duct [3]. However, breast milk (BM) contains the appropriate nutritional components and digestive enzymes, but also immunologically effective and protective components like antibodies, secretory Immunoglobulin A (slgA), lysozyme, oligosaccharides, growth factors, neuregulin-4, lactoferrin and cellular components which augment the infants active host defenses [4]. Completely weaning children with chylothorax off breast milk means the loss of these components, which are essential for an enhanced neurological development [5]. Moreover, special milk formulas are not freely available in developing countries and are far too expensive, so that the use of fat-free human milk was already reported by Chan in 2007 [6]. The use of skimmed milk was found to be equivalent or even better to specialized formulae and can be a therapeutic option [7]. Octreotide, a synthetic long-acting analogue of somatostatin, is an additional strategy in the treatment of chylothorax, because it inhibits lymphatic fluid production by acting on somatostatin receptors in the splanchnic vessels [8].

A definitive guideline for medical management and therapeutic dietary intervention of congenital chylothorax is lacking. Considering the importance of breast milk feeding, especially in preterm infants [9], we tried to use skimmed breast milk (SBM) to avoid its discontinuation. We report our experience with the early, but careful successive introduction of long-chain fatty acids (LCFA) in form of skimmed breast milk after resolution of chylothorax with different outcomes in neonates with congenital chylothorax.

## Methods

Neonates diagnosed with congenital chylothorax and treated between 2013 and 2020 at Innsbruck Medical University Hospital (*n* = 6) were eligible for this report.

In three of the six patients, we used skimmed breast milk for enteral nutrition to provide high protein and low-fat diet. The re-establishment of feeds of the other three patients was via medium-chain-triglycerides based milk formulas.

**Fig. 1:**
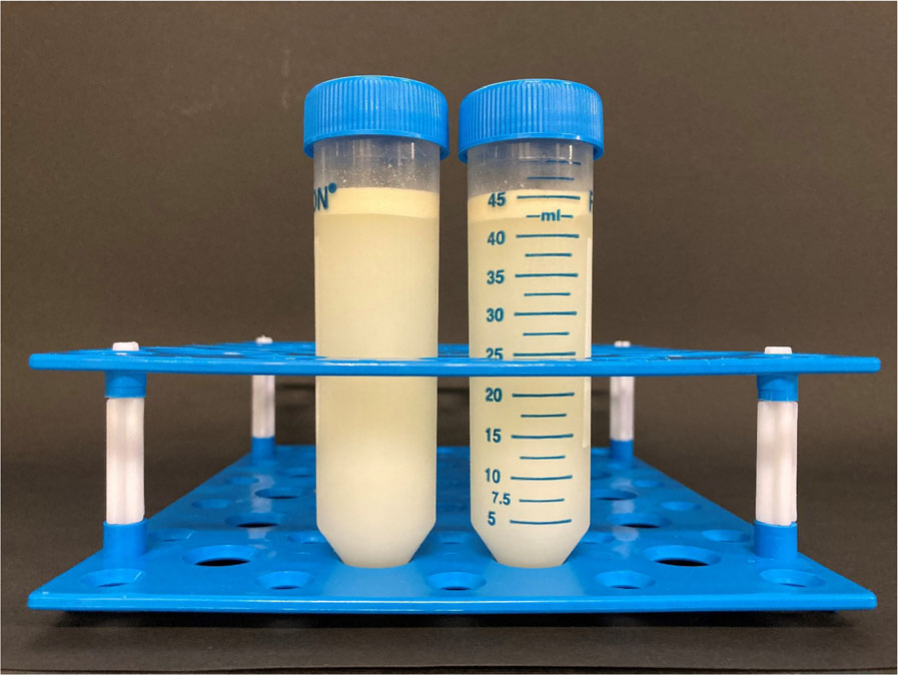
Clearly visible separation of fat and fat-free fraction after centrifugation

Skimmed milk is defined as the nearly fat-free fraction of breast milk and can be produced via centrifugation or spontaneous separation. We decided to use a centrifugation-based method because it was shown to be more effective at separating fat in human milk. BM was expressed and stored at 0°- 4 °C in a refrigerator for maximal 24 h until it was processed at the local human milk bank. Expressed breast milk (EBM) was then transferred to sterile conical centrifuge tubes (Falcon™ 50 ml polypropylene conical tubes) and centrifuged for 10 min at 2000 rpm and 5 °C (Centrifuge 5810R®, Eppendorf). Separation of the fat fraction and the skimmed portion was clearly visible (shown in Fig. 1). Aspiration of the fat-free fraction was performed with a syringe and an attached sampling straw. Skimmed breast milk was either stored at 0°- 4 °C and fed within 24 h from the time BM was expressed or stored at ≤ − 18 °C for at least 24 h and used thereafter. After thawing, SBM was used within 24 h and not frozen again [10].

## Results

In a time period of 8 years, we identified a total of six infants with congenital chylothorax. All six neonates were symptomatic at birth and showed severe respiratory distress. They needed stabilization of the respiratory situation via chest tube drainage and respiratory support. The decision, whether non-invasive (CPAP), conventional or high frequency ventilation has been employed, depended on the severity of respiratory failure.

The dietary treatment and trial of octreotide therapy varies among our patients and included the following steps:

### Patient 1 (36 + 4 weeks, 3080 g)

A diet with Monogen® was started on the first day of life (DOL). However, even with total parenteral nutrition (TPN), the chyle amount did not decrease. On day 16, therapy with somatostatin was initiated and slowly increased to a peak dose of 5 μg/kg/h. After clinical improvement somatostatin was reduced and discontinued on day 26 (cumulative dose 2.83 mg). Chyle amount decreased gradually and enteral nutrition with Monogen® was restarted on day 18, reaching an amount of 150 ml/kg/day on day 23, when parenteral nutrition was discontinued. The infant was discharged at 40 + 4 weeks and 3750 g (50th percentile) on the 27th DOL on Monogen® and LCFA and vitamin substitution once a week till she was transitioned to full-fat formula nutrition on day 77. Breast milk was never fed.

### Patient 2 (35 + 0 weeks, 3200 g)

After 1 week of TPN, Basic F® was introduced. On full enteral nutrition, recurrence of pleural effusion and respiratory deterioration led to the introduction of therapy with somatostatin with a maximal dose of 10 μg/kg/h on the 14th DOL. Nutrition with Basic F® was continued again and diet was gradually changed to Monogen® to introduce some LCFA on day 29. Somatostatin was discontinued on day 41 (cumulative dose 15.65 mg). The patient was discharged on Monogen® and LCFA and vitamin substitution once a week (until the 84th DOL) on day 43 at 41 + 0 weeks and 4415 g (90th percentile). Breast milk was gradually introduced at the 56th DOL.

### Patient 3 (32 + 5 weeks, 2200 g)

TPN was performed until the 13th DOL. Additionally, therapy with somatostatin was commenced on day 3, reaching a maximal dose of 10 μg/kg/h on day 5 and discontinued on day 32 (cumulative dose 12.6 mg). After 2 weeks Basic F® was introduced and replaced by Monogen® 1 week later. LCFA and vitamins were substituted once a week (until the 54th DOL). The patient was dismissed on day 43 at 38 + 5 weeks and 2885 g (10th percentile). Breast milk was gradually introduced at the 47th DOL.

### Patient 4 (36 + 3 weeks, 2520 g)

After 1 week of TPN, effusion amount decreased and enteral nutrition with Basic F® was introduced. Subsequently, effusion amount and tachydyspnoe worsened again. For this reason, somatostatin was started on day 16 at a maximum dosage of 15 μg/kg/h and discontinued on the 56th DOL (cumulative dose 32.9 mg). Moreover, parenteral nutrition was introduced for another 2 weeks. After stabilization, Basic F® was restarted on day 35 and converted to skimmed milk within 2 weeks. LCFA and vitamins were substituted once a week till the 57th DOL. Under this regime, weight gain was adequate and chylothorax did not recur. From the 60th day on, the patient was fully breastfed and was dismissed on the following day at 45 + 0 weeks and 4105 g (35th percentile).

### Patient 5 (35 + 4 weeks, 2700 g)

This was a female neonate with confirmed *RAF1* mutation (Noonan syndrome). Ligation of a persistent ductus arteriosus Botalli at the age of 28 days revealed congenital lymphatic system malformations and subsequently a chylothorax. Afterwards the patient was on parenteral nutrition for 5 days, and somatostatin was additionally given at a maximal dose of 10 μg/kg/h for 24 days (cumulative dose 8.31 mg). Since day 42, when pleural effusions resolved, the patient was fed with skimmed breast milk and reached full enteral feeding 10 days later. However, the baby’s condition was complicated due to the underlying Noonan syndrome and two weeks later the mother wanted to stop to express breast milk, so skimmed milk was changed to Basic F® and LCFA and vitamin substitution once a week. Full-fat formula nutrition was introduced from the 73rd day on with one relaps of pleural effusion, so full transition was finally on day 116. The patient was dismissed on day 128 at 53 + 5 weeks and 4950 g (1st percentile).

### Patient 6 (37 + 3 weeks, 2920 g)

A TPN and a therapy with somatostatin (cumulative dose 14.38 mg) was performed for at least 2 weeks. When the effusion resolved, feeding with skimmed milk was started. However, 2 days later we observed a re-accumulation of pleural fluid so we had to change to Basic F® and LCFA and vitamin substitution once a week. After that, the enteral nutrition was without any problems. The patient was dismissed on day 27 at 40 + 4 weeks, 3680 g (50th percentile).

**Fig. 2:**
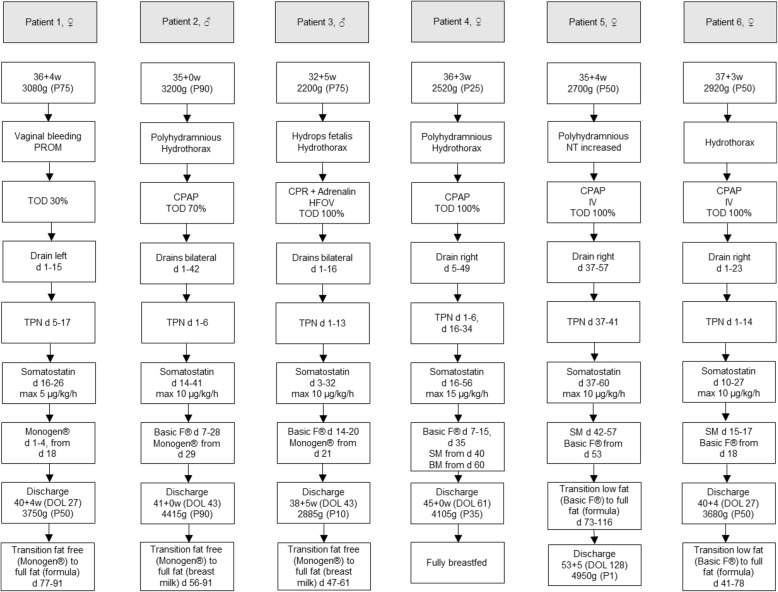
Patient characteristics and CC management at the NICU Innsbruck

A summary of patient characteristics and CC management at the NICU Innsbruck is given below (shown in Fig. 2).

## Discussion

**Fig. 3:**
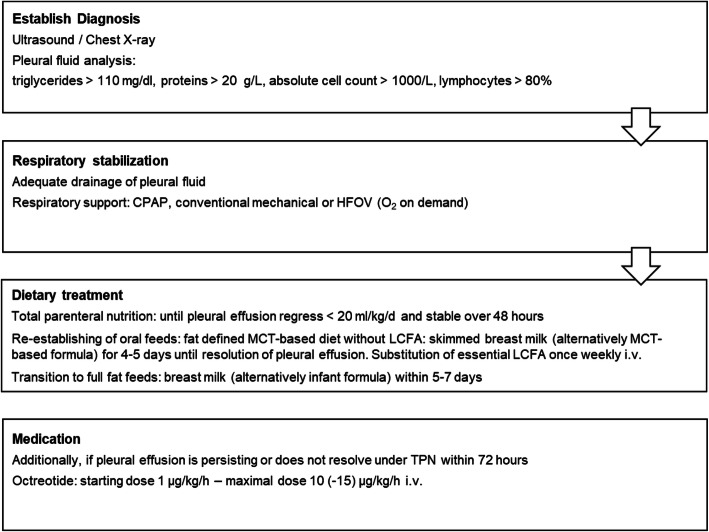
Recommendation for the management of CC

Congenital chylothorax is an uncommon but serious entity in neonates. Due to the rarity of this disorder a universal consensus on management of CC is unavailable and current dietary treatment recommendations are based on individual case reports or case series [11]. Since 2013, over a period of 8 years, we have identified and treated a total of six infants with congenital chylothorax. After literature review and analysis of our own experience, we propose an algorithm for the treatment of this entity with focus on dietary management (see in Fig. 3).

The accumulation of lymphatic fluid with high levels of triglycerides (> 110 mg/dl), proteins (> 20 g/L), and lymphocytes (> 80% of cells) implicates large losses of nutrients and immune cells and puts patients at risk of malnutrition and impairs their immune system [12]. So, when dealing with the effusion, nutritional management is a key issue and a balance is needed between achieving sufficient caloric intake and minimizing chyle production. Moreover, a rapid conversion to regular alimentation (preferably breast feeding) is especially important for preterm infants as the growing brain is strongly dependent on the supply of balanced fatty acid nutrition [13]. Enteral feeding with MCT bypasses the intestinal lymphatic system, as they are absorbed directly into the portal venous system. Thus, therapy of chylothorax calls for MCT-based nutrition with adequate LCFA supplementation, as they are needed as essential part and precursors for membranes and other metabolic processes. This reduces the chyle flow. However, even the intake of sterile water can stimulate chyle flow by 20% [14]. Therefore, total parenteral nutrition should be applied until the pleural effusions have resolved [15]. Thereafter, diet needs to be cleared of long-chain fatty acids over several weeks or even months. Thus, chylothorax is considered an absolute contraindication for breastfeeding as human milk has a high long-chain fatty acid content [16]. The dietary management of our six patients consisted of TPN, which was performed until pleural effusions resolved (mean 12.6 days). To avoid a prolonged parenteral nutrition and its possible adverse effects including a high risk of sepsis, nutritional support was provided in the form of adequate caloric intake and enteral feedings were gradually introduced using a low-fat formula. Basic F® was applied to all babies, except patient 1, who immediately received Monogen®. LCFA like Clinoleic acid 20% (2 g/kg) and vitamins like Vitalipid® (4 ml/kg – max 10 ml) and Soluvit® (1 ml/kg) were substituted once a week. In further course the introduction of a medium-chain triglyceride (MCT)-based formula containing some LCFA (16% of energy) was attempted. About 80% of infants with chylothorax respond to conservative dietary management [17]. Nevertheless, chylothorax did not resolve in any of our patients under this regime, and therefore treatment with somatostatin was started additionally. The initial dose varied from 0.5 to 1 μg/kg/h and the maximum dose applied was 15 μg/kg/h. In human milk somatostatin is found in high concentrations, thus contributing a direct beneficial effect toward reducing lymph production when human milk is continued in infants with chylothorax [18]. If breast milk feeding is continued, medication with octreotide can be avoided and uncommon but potentially life-threatening side-effects like necrotizing enterocolitis (NEC) [19], pulmonary hypertension or aggravation of bronchopulmonary dysplasia (BPD) may possibly be prevented [20].

Breast milk has proven to have beneficial effects and is strongly recommended for all infants, particularly for preterm infants because of its nutritional, immunologic and psychosocial advantages [21]. Abstaining from breast milk causes infants to not be optimally protected against serious diseases of preterm infants like gastrointestinal infections or NEC. Furthermore, a chylothorax diagnosis can be frustrating for parents, especially for those who had intended to provide breast milk as the primary form of nutrition for their infant. Consequently, the mother-child interaction may suffer because of the missing physical contact. Against this background, we aimed to successfully introduce a dietary regimen of fat-modified breast milk that can provide the immune, nutritional and bonding benefits of breast milk without exacerbating chylous effusions. Accordingly, in Patients 4, 5 and 6 we administered skimmed breast milk instead of an MCT-based formula after resolution of chylothorax on day 40, 42 and 15, respectively. The outcome was : patient four was associated with a favourable outcome, meaning no recurrence of pleural effusion, adequate weight gain and a contented mother. The condition of the fifth patient was difficult due to a diagnosed Noonan syndrome . Two weeks after the introduction of skimmed breast milk the mother wanted to stop to express breast milk, so nutrition was changed to Basic F®. Patient six had a relapse of pleural effusion after the administration of skimmed milk and was therefore transitioned to Basic F® again. The literature shows reports on several cases of successful use of fat-free (skimmed) breast milk [6, 22–25]. More and more clinics worldwide are introducing skimmed breast milk instead of special fat-free nutrition. This can be provided via centrifugation (at minimum 2500 rcf for 15 min; which is approx. 3000–3500 rpm depending on the centrifuge) or by placing the milk in the refrigerator and leaving it undisturbed until the fat fraction and the transparent, fluid skimmed portion spontaneously separate (after about 4-6 hours), so that it is also practicable after discharge. Skimmed milk has had the long-chain fatty acids removed (fat content is less than 0.1%), is lower in calories, essential fatty acids and fat-soluble vitamins, which have to be parenterally replaced. However, it retains levels of electrolytes, protein and lactose that are similar to those of normal breast milk, and it can thus be assumed that the immunologically protective components of breast milk that are contained in proteins are largely preserved during the procedure [6]. However, in future studies we plan to analyze EBM for creamatocrit, a clinical technique for estimating concentration and energy value of human milk. If the fat content is higher than acceptable limits its application has to be re-evaluated, because a relapse of pleural effusion – like in our last patient – is possible [26]. It is also important to mention the commitment of the mother - who has to express her breast milk - which is required for skimming and not always possible, like in patient five with the Noonan syndrome.

## Conclusion

The first-line therapy of chylothorax is a combination of respiratory stabilization and dietary modification. The use of skimmed breast milk is advisable in CC and feasible by means of a simple milk defatting procedure. It offers benefits to mothers who wish to resume breast feeding after resolution of chylothorax and has proven positive effects, above all in preterm infants as optimal nutrition with protective components superior to formula feeding. However, the nutritional analysis of the skimmed milk and the correlation to a re-accumulation of pleural fluid remains a question to be answered. Furthermore, a future multicenter study in a larger number of patients will be helpful and necessary to determine the applicability of skimmed breast milk in the treatment of CC and allow the development of an international management guideline for CC.

## Data Availability

The datasets during and/or analyzed during the current study available from the corresponding author on reasonable request.

## Abbreviations

BPD: Bronchopulmonary dysplasia
BM: Breast milk
CC: Congenital chylothorax
CPAP: Continuous positive airway pressure
CPR: Cardiopulmonary resuscitation
CTD: Chest tube drainage
DOL: Day of life
EBM: Expressed breast milk
HFOV: High-frequency oscillation ventilation
IV: Invasive ventilation
LCFA: Long-chain fatty acids
LCT: Long-chain triglycerides
MCT: Medium-chain triglycerides
NICU: Neonatal intensive care unit
NT: Nuchal translucency
PROM: Premature rupture of membranes
SBM: Skimmed breast milk
TOD: Total oxygen demand
TPN: Total parenteral nutrition
